# Mothers' Own Recollections Of Being Parented And Risk Of Offspring Depression 18 Years Later: A Prospective Cohort Study

**DOI:** 10.1002/da.22174

**Published:** 2013-09-19

**Authors:** Liam Mahedy, Jon Heron, Lexine A Stapinski, Rebecca M Pearson, Jonathan Evans, Carol Joinson, Lucy Bowes, Glyn Lewis

**Affiliations:** Centre for Mental Health, Addiction and Suicide Research, School of Social and Community Medicine, University of BristolBristol, UK

**Keywords:** adolescence, ALSPAC, depression, maternal bonding

## Abstract

**Background:**

Although the relationship between maternal bonding and risk of offspring depression has been demonstrated, it is unclear whether this risk exists for subsequent generations. This study examines the association between maternal reports of her own mother's parenting and later risk of depression in offspring at age 18.

**Method:**

This study is based on data from the Avon Longitudinal Study of Parents and Children. Mothers enrolled in the study, completed the Parental Bonding Instrument to provide an assessment of how they were parented by their own mothers up to the age of 16. Offspring depression was assessed at age of 18 using the Clinical Interview Schedule-Revised. The sample comprised 10,405 respondents who had completed the Parental Bonding Instrument during the antenatal period. Results were adjusted for grandmother's history of depression, maternal depression, and a range of socioeconomic variables.

**Results:**

A one standard deviation increase in mothers’ perceived lack of care in their own childhood was associated with a 16% increase in the odds of offspring depression at age 18 (odds ratios = 1.16, 95% confidence intervals = [1.04, 1.30]). This effect remained following adjustment for potential confounders (odds ratios = 1.14, 95% confidence intervals = [1.02, 1.27]). There was no evidence for an association between overprotection and offspring depression.

**Conclusions:**

This study is consistent with the hypothesis that sensitive caregiving is important to future risk of depression across generations. Preventative interventions could be aimed at promoting positive parenting practices, which may help to reduce the risk of depression in subsequent generations.

## INTRODUCTION

Depression is among the most prevalent of psychiatric disorders in adolescents. Since mental health disorders commonly begin in childhood and adolescence,^1^ this is an important period for understanding the developmental origins of these disorders. By 2030, depression will rank first in high-income countries among disorders contributing to global disease burden.^2^ Given the social^3^ and economic^4^ costs of depression, it is becoming increasingly important to identify and establish strategies that might prevent the onset of depression.

One area of research that has gained considerable attention is the role of the parent–child relationship as it is thought to provide the platform for socioemotional development and regulation in the child.^5^,^6^ Focusing on parenting practices, the Parental Bonding Instrument (PBI)^7^ is the measure of choice for many researchers as it is a well-validated measure^8^ and is quick and easy to administer.^9^ There is consistent evidence that parenting behaviors, as measured by the PBI, are related to offspring depression.^10^ Similarly, research using animal models has demonstrated that lack of early maternal care influences vulnerability for animal models of stress.^11^

Although the relationship between parenting and risk of offspring depression has been established, it is still not known whether the risk for depression may persist into subsequent generations. Intergenerational transmission of parenting practices has been well established throughout the literature with previous research focusing on the transmission of negative/harsh parenting^12^ and warm/sensitive parenting across generations.^13^,^14^ Consequently, mothers who experience a lack of care in their own childhood may show a lack of care to their own children, reinforcing a cycle of increased risk of depression from generation to generation.

Research investigating the influence of parenting has been hampered by several methodological problems including method variance, that is, reports of parenting and offspring's depressive symptoms are both derived from a single reporter,^15^ reliance on small sample sizes,^16^ cross-sectional design,^15^ and relatively short follow-up periods of prospective studies.^17^,^18^ A strength of the current study is the availability of data on a range of confounding variables assessed during the antenatal period (e.g., maternal age, maternal concurrent depression, and grandmother's history of depression) that have been shown to be associated with maternal recollections of being parented^19^ and offspring depression.^20^

The current study, based on a large UK cohort, examines the effect of mother's own recollections of being parented (assessed in the antenatal period) and later risk of offspring self-reported depression (assessed in the offspring at age 18). Focusing specifically on the relationship between mothers’ recollections of being parented and risk of offspring depression at age 18, addresses a number of these methodological limitations. There is no research in large prospective cohorts examining whether a mother's own experiences of being parented by her own mother impacts upon risk of depression in her offspring.

## METHOD

### SAMPLE

Data from the Avon Longitudinal Study of Parents and Children (ALSPAC) consist of 15,247 pregnant mothers residing in the former Avon Health Authority in the southwest of England, having an estimated date of delivery between April 1, 1991 and December 31, 1992. The core sample for these analyses was limited to singletons who survived to 1 year (*n* = 13,617). Please note that the study website contains details of all data that is available through a fully searchable data dictionary.^21^ Ethical approval for the study was obtained from the ALSPAC Ethics and Law Committee and the Local Research Ethics Committees. For further details on the cohort profile, see.^22^

From the sample of 13,617 respondents, 1,169 (8.5%) respondents did not return the questionnaire, while an additional number of 622 (5%) did not complete any of the PBI items. A further 1,421 (10%) respondents were omitted from the sample as they completed the PBI during the postnatal stage. Our core working sample is defined by the 10,405 respondents who had completed at least one item of the PBI during the antenatal period. Of these, 9,223 (88.7%) respondents had complete information on the PBI.

### MEASURES

#### Parental Bonding Instrument (PBI)

The PBI^7^ is a 25-item self-rating instrument asking individuals, over the age of 16, about their own experiences of being parented up to the age of 16. In our study, the PBI was completed by mothers throughout the antenatal period. Previously in the literature, *care* and *protection*/*control* were the two original parenting dimensions.^7^ More specifically, 12 care items are comprised warmth, empathy, and affection while 13 protection/control items comprised overprotection, control, and prevention of independence versus autonomy. Although there has been general consensus about the *care* factor, there is less certainty about *overprotection*. Previous studies suggest that overprotection could be divided into two factors to incorporate *protectiveness* and *authoritarianism*.^23^

The version used in the ALSPAC study was adapted by^24^ and contained a number of further adaptations. During the piloting stage, three items were omitted (*Did not want me to grow up, made me feel I wasn't wanted, and tried to make me feel dependent on her*) from the original version as they duplicated existing items. Thirteen items were given responses as *never, sometimes*, or *usually*, while the remaining nine items were given dichotomous *yes/no* response options. Due to many small cell counts in the uncollapsed data, all items were dichotomized in order to help with estimation.

#### Adolescent Depression

Offspring adolescent depression data from 4,566 adolescents who completed the Clinical Interview Schedule-Revised (CIS-R)^25^ at age 18 were examined. The CIS-R is a self-administered computerized interview, which derives a diagnosis of depression for algorithms based on ICD-10 criteria for depression. A binary *yes/no* variable indicating a primary diagnosis of major depression was the outcome measure for this study.

#### Confounding Variables

Adjustment was made for potential confounding variables recorded by self-report postal questionnaires at 32 weeks gestation. These included maternal age at birth; maternal concurrent depression using the Edinburgh Postnatal Depression Scale (EPDS),^26^ maternal education (<O level, O level, ≥A level); maternal social class (ranked from high to low at five intervals); and grandmother's history of depression (*yes/no*) measured at 12 weeks gestation. Figure 1 (online supplement) shows a timeline of variables used in the analysis, focusing on both maternal and offspring self-reports.

### STATISTICAL ANALYSIS

Statistical analysis was conducted using Mplus v7.^27^ Initially, confirmatory factor analysis (CFA) was used to establish the factor structure of the PBI. CFA was utilized using the WLSMV (mean and variance adjusted weighted least squares) estimator,^28^ as this estimator performs best in the CFA modeling of categorical data.^29^ In assessing the fit of the CFA model, four fit statistics were used (1) chi-square statistic (χ^2^); (2) root-mean-square error of approximations (RMSEA);^30^ (3) comparative fit index (CFI)^31^; and (4) Tucker–Lewis fit index (TLI).^32^ Goodness of fit was determined in accordance with^33^ and was indicated by a χ^2^ < 0.05, CFI and TLI values of over 0.95, and RMSEA of less than 0.06. Multiple indices were used as they provide a more comprehensive evaluation of model fit. Logistic regression was used to examine the relationship between the PBI and offspring adolescent depression. Odds ratios (OR) and their 95% confidence intervals (CI) were calculated using logistic regression.

#### Missing Data

The outcome variable at 18 years (*n* = 4,566) comprised just under half of the core working sample (*n* = 10,405). Since using complete case analysis and not taking missing data into account can result in biased estimates,^34^ an approach was taken to impute missing data using multivariate imputation by chained equations via the *ice* method^35^ in Stata 12. This method is based on the assumption that data are missing at random conditional on the observed covariates and outcomes. A number of auxiliary variables predictive of incomplete variables and/or missingness were included in the model; these included sociodemographic variables and reports of depression at additional time points. To ensure an accurate representation of the data, 100 imputed datasets were derived, each entailing 20 cycles of regression switching.

Sensitivity analyses examined the impact of various sources of missing data (online Supporting Information Table 1). The first model comprised the complete cases sample (*n* = 3,171). Imputation was then carried out on three further models, Model 1: *n* = 4,566 (information available for outcome measure, but an incomplete set of exposure and confounding variables); Model 2: *n* = 9,223 (complete information on exposure, and incomplete data on confounding and outcome variables); and Model 3: *n* = 10,405 (information on exposure measure, but incomplete data on outcome and/or confounding variables).

## RESULTS

CFA (Table 1) was conducted using the core working sample of 10,405 respondents who completed the PBI during the antenatal period, mean age = 28.3, standard deviation = 4.8. For our purposes, two items were omitted from the analysis. Item 15: *babied by mother* was omitted due to a low standardized factor loading of 0.28 on the overprotection factor while Item 20: *allowed by mother to go out anytime* was omitted as it was highly correlated with Item 21: *was overprotective of me*.

**Table 1:** Confirmatory factor analysis of the PBI (standardized coefficients)

Item	Yes (%)	Lack of care
1. Spoken to warmly by mother	79.1	0.886
2. Helped as needed by mother	80.6	0.908
4. Apparent coldness from mother^a^	4.9	0.764
5. Problems seem understood by mother	51.0	0.835
6. Affection by mother	70.4	0.870
10. Felt unwanted by mother^a^	3.8	0.634
11. Things talked over by mother	55.3	0.829
13. Praised by mother	52.4	0.812
14. Mother enjoyed talking things over	80.7	0.894
15. Frequently smiled at by mother	89.6	0.893
17. Needs understood by mother	78.3	0.921
18. Upsets comforted by mother	86.4	0.886
	Yes (%)	Overprotection
3. Allowed by mother to do as like^a^	42.0	0.809
7. Control attempted by mother	25.4	0.594
8. Privacy invaded by mother	7.9	1.036
9. Allowed own decisions by mother^a^	45.3	0.700
12. Allowed freedom by mother^a^	36.3	0.752
16. Babied by mother	——	——
19. Felt helpless without mother	15.6	0.542
20. Allowed by mother to go out anytime	——	——
21. Overprotective mother	20.6	0.473
22. Allowed by mother to dress as liked^a^	42.4	0.445

a Items were reversed scored.

Table 1 reports the standardized factor loadings of the PBI. The correlation between the two factors was .62. The results from a two factor model indicate factor loadings ranging from .63 to .92 for the *care* factor and from 0.45 to 1.04 for the *overprotection* factor. A two-factor model provided an adequate description of the data (χ^2^ = 7308.819, *df* 207, *P* = .001; RMSEA = 0.061 (90% CI = [0.060, 0.062]), CFI = 0.957; and TLI = 0.951).

Overall, 4,566 adolescents completed the CIS-R at 18 years of age. Fifty-six percent of the sample who completed the CIS-R at 18 years were female. A primary diagnosis of ICD-10 depression was given for 360 (8%) of offspring respondents. Of these 270 (75%) of the sample who were classified as having a diagnosis of depression at 18 years were female.

From the sample who completed the CIS-R (*n* = 4,566), 3,521 (77%) respondents also had available data on all items of the PBI. Demographics for the complete cases sample compared to the rest of the core sample are provided in Table 2. Respondents from the complete cases sample were older, better educated, had lower incidence of maternal depression, and were of higher social class.

**Table 2:** Demographic variables for the sample with and without outcome data at 18 years

	CIS-R 18 years *n* = 3,171	CIS-R missing *n* = 6,884	χ^2^ (*df*)	*P*-value
Maternal education				
<O level	463 (14.6%)	1,737 (25.2%)	482.30 (3)	.000
O level	1,087 (34.3%)	2,298 (33.4%)		
≥A level	1,490 (47.0%)	1,966 (28.6%)		
Missing	131 (4.1%)	883 (12.8%)		
Maternal depression				
Yes	375 (11.9%)	1,015 (14.7%)		
No	2,750 (86.7%)	5,583 (81.1%)	125.52 (2)	.000
Missing	46 (1.4%)	286 (4.2%)		
Grandmother's depression				
Yes	604 (19.0%)	1,410 (20.5%)		
No	2,567 (81.0%)	5,474 (79.5%)	2.28 (1)	.131
Missing	0	0		
Maternal social class				
High 1	230 (7.3%)	240 (3.5%)	387.49 (6)	.000
2	1,039 (32.8%)	1,507 (21.9%)		
3	1,310 (41.3%)	2,783 (40.4%)		
4	214 (6.8%)	542 (7.9%)		
Low 5	32 (1.0%)	125 (1.8%)		
Missing	346 (10.9%)	1,687 (24.5%)		
	Mean (S.D.)	Mean (S.D.)	*t* (*df*)	*P*-value
Maternal age	29.31 (4.50)	27.77 (4.89)	15.50	.000

There was strong evidence of an association (*P* = .007) between maternal lack of care and offspring depression at age of 18 (Table 3). For a one standard deviation increase of reporting maternal lack of care in childhood, there was a 1.16 (95% CI = [1.04, 1.30]) increase in the odds of offspring depression at age of 18. This relationship was not substantially affected after adjusting for a range of confounding variables (OR = 1.14; 95% CI = [1.02, 1.26]). There was no evidence to suggest that recollection of overprotection during childhood was associated with risk of offspring depression 0.99 (95% CI = [0.88, 1.11]). This relationship was not substantially affected after adjusting for a range of confounding variables (OR = 1.01, 95% CI = [0.90, 1.11]). Results from pre- and postimputation models were consistent across all analyses of varying sample sizes (online Supporting Information Table 2).

**Table 3:** Standardized regression coefficients for offspring depression and maternal bonding

	Unadjusted model OR (95% CI)	Adjusted model OR (95% CI)	*P*-value
Lack of care	1.16 (1.04, 1.30)	1.14 (1.02, 1.26)	.007
Overprotection	0.99 (0.88, 1.11)	1.01 (0.90, 1.11)	.624

Model adjusted for maternal age, maternal social class, maternal education, maternal depression, and grandmother's history of depression.

## DISCUSSION

Our findings demonstrated an association between maternal experiences of being parented and risk of offspring depression at 18 years of age. Offspring of mothers who reported they received a lack of care from their own mothers in childhood had a 14% increase in odds of experiencing depression at age 18. This relationship persisted after controlling for other variables that have been shown to be related to maternal reports of being parented and risk for adolescent depression, for example, maternal age, depression, education, and social class. In comparison, there was no evidence that the odds of offspring depression were increased in 18-year-old offspring of mothers who endorsed items pertaining to overprotection in the PBI.

### STRENGTHS AND LIMITATIONS

The present study has a number of strengths, including data from a long follow-up period between the mother's antenatal assessment of being parented and depression in offspring at age of 18. Having self-reports of maternal recollections of being parented and depression in the offspring addresses the issue of method variance. This study is further strengthened by the large sample size of over 10,000 respondents who completed the PBI during the antenatal period. Furthermore, since maternal-reported parenting was assessed prior to the infant being born, it eliminates the potential for infant temperament biasing the PBI rating.

Given the longitudinal nature of this study, we can exclude the possibility of reverse causality, as adolescent depression cannot influence the manner in which mothers bond with their own mothers in childhood. Although the strength of the relationship is modest, it does demonstrate an association over an 18-year period. Furthermore, the inclusion of a range of possible confounding variables was shown not to alter the strength of the relationship between exposure and outcome variables.

A number of limitations are also noted. First, a limitation common to most longitudinal studies is sample attrition. Adolescents who attended the clinic at age of 18 were more likely to come from families that had lower incidence of maternal depression, were better educated, and of higher social class. To address biases that may be present due to response attrition, a number of sensitivity analyses using imputed data were conducted. The findings were consistent across analyses suggesting a robust pattern of results.

Although retrospective questionnaires have been widely used throughout the literature, criticism surrounds the use of self-report methods. For example, one concern is that depression can bias memory toward negative events.^36^ Despite these criticisms, two recent studies have examined the stability of the PBI over a 20-year period. For example, one study found that recollections of one's parental environment is not substantially influenced by gender, depression history, and life experience and that subject's perceptions do not shift with fluctuations in depressed mood or neuroticism level.^37^ More recently,^19^ it has been demonstrated that although the PBI is sensitive to sample characteristics, time and mood fluctuations, this sensitivity does not appear to significantly bias the long term stability of the instrument.

A further limitation relates to the sole focus on maternal bonding, while the role of fathers was not examined. That being said, the majority of previous research has demonstrated a greater association with psychopathology for maternal bonding as compared to paternal bonding.^15^ Traditionally, mothers are typically the primary emotional caregiver and their parenting behavior may be more influential than fathers.^38^

## IMPLICATIONS

Expanding on previous research that found the early caregiving environment to be a risk factor for mental health problems,^15^,^18^ our findings highlight the intergenerational consequences of suboptimal caregiving. In doing so, they extend the findings from previous studies^15^,^18^ who found that maternal lack of care was a risk factor for adult psychopathology, by demonstrating that experiencing a lack of maternal care in childhood places subsequent generations at risk of depression.

Although this study does not suggest that parenting practices in one generation are repeated in the next, our findings reveal that a grandmother's parenting behavior influences their grandchild's risk of depression in adolescence. Although maternal parenting practices in relation to risk of offspring depression were not examined in this study, it is possible that mothers who experience a lack of care in their own childhood show a lack of care to their own children. The specific mechanisms underlying this intergenerational transmission may be clarified in future research.

Although we cannot completely rule out the role of shared genetic vulnerability, the findings are shown to be independent of grandmother and maternal concurrent depression that were included as possible confounding variables. Research using animal models further demonstrates that lack of early maternal care acts as a risk factor in the regulation of emotional and cognitive responses to stress,^39^ which may influence vulnerability for mood disorders.^11^ Francis et al.^40^ further demonstrated using a cross-fostering study (young rat pups adopted by a caring mother from a neglecting mother and vice versa) that this effect was independent of genetic influences, as infants’ tolerance to stress was consistent with the caring behavior of their adopted rather than biological mother. These findings indicate that parenting styles may influence the development of emotion regulation capacities and the risk of poor regulatory capacities may persist across subsequent generations.

## CONCLUSION

The findings add to the understanding of the relationship between the long-term effects of the early caregiving environment and adolescent depression and may have implications for policy making and parenting practices.^41^ For example, women who have been identified as experiencing a lack of care in their own childhood are at greater risk of having offspring who develop depression during adolescence. In this regard, potential mothers can be identified at an early age and targeted for early intervention. Given the intergenerational nature of these findings, it is suggested that the promotion of positive parenting programs could reduce the risk of depression across generations in the long term.
