# Supplementary material for: Mothers' Own Recollections Of Being Parented And Risk Of Offspring Depression 18 Years Later: A Prospective Cohort Study
Source: Depress Anxiety. 2013 Sep 19;31(1):38–43. doi: 10.1002/da.22174 (PMC3992906; doi:10.1002/da.22174)
Supplement: Supplementary file 1 [file da0031-0038-sd1.doc]

**Online Supplement**

| **Exposure**  Bonding with own mother (PBI)  **Outcome**  Diagnosed Depression (CIS-R)      Gestation  12 wks Birth Child Age 18 yrs  32 wks  **Key** Informant:  Mother  Offspring  **Covariates**  Grandmother history of depression  **Covariates**  Maternal Age  Maternal education  Maternal S class  Maternal depression (EPDS |
| --- |

Figure 1. Schedule for exposure, confounding and outcome measures

**Table 1.** *Demographic variables for the sample with and without outcome data at 18 years*

|  | **Exposure and Outcome data** | **Outcome data** | **Complete exposure data** | **Core sample** |
| --- | --- | --- | --- | --- |
|  | *n* = 3,171 | *n* = 4,566 | *n* = 9,223 | *n* = 10,405 |
| Depression 18 |  |  |  |  |
| No | 2,936 (92.5%) | 4,206 (92.1%) | 2, 932 (31.7%) | 3,251 (31.2%) |
| Yes | 235 (7.5%) | 360 (7.9%) | 239 (2.5%) | 270 (2.6%) |
| Missing | 0 | 0 | 6,052 (65.6%) | 6,884 (66.2%) |
| Maternal Education |  |  |  |  |
| <O level | 577 (16.7%) | 530 (11.6%) | 1,955 (21.2%) | 2,267 (21.8%) |
| O level | 1,094 (34.7%) | 1,196 (26.2%) | 3,189 (34.6%) | 3,494 (33.6%) |
| ≥A level | 1,500 (46.6%) | 1,633 (35.8%) | 3,245 (35.2%) | 3,598 (34.6%) |
| Missing | 0 | 1,207 (26.4%) | 834 (9.0%) | 1,046 (10.1%) |
| Grandmother Depression |  |  |  |  |
| No | 2,571 (81.0%) | 2,844 (62.3%) | 7,368 (79.9%) | 8,318 (80.0%) |
| Yes | 600 (19.0%) | 677 (14.8%) | 1,855 (20.1%) | 2,087 (20.0%) |
| Missing | 0 | 1,045 (22.9%) | 0 | 0 |
| Mother Depression |  |  |  |  |
| No | 2,768 (88.4%) | 3,046 (66.1%) | 7,612 (82.5%) | 8,510 (81.8%) |
| Yes | 381 (12.1%) | 419 (9.1%) | 1,235 (13.4%) | 1,434 (13.8%) |
| Missing | 44 (0.5%) | 1,101 (24.8%) | 376 (4.1%) | 461 (4.4%) |
| Maternal social class |  |  |  |  |
| High 1 | 230 (7.3%) | 251 (5.5%) | 450 (4.9%) | 491 (4.7%) |
| 2 | 1,039 (32.8%) | 1,153 (25.3%) | 2,397 (26.0%) | 2,659 (25.6%) |
| 3 | 1,310 (41.3%) | 1,442 (31.6%) | 3,845 (41.7%) | 4,225 (40.6%) |
| 4 | 214 (6.75) | 239 (5.2%) | 683 (7.4%) | 781 (7.5%) |
| Low 5 | 32 (1.0%) | 36 (0.8%) | 139 (1.5%) | 161 (1.6%) |
| Missing | 346 (10.3%) | 1,445 (31.7%) | 1,709 (18.5) | 2,088 (20.1%) |
| Mean Maternal Age (S.D.) | 29.3 (4.5) | 29.3 (4.5) | 28.32 (4.8) | 28.3 (4.8) |

***Table 2.*** Comparison of relationship between PBI and offspring depression using different imputation samples

|  | **Exposure and outcome data^1^** | **Outcome data^2^** | **Complete exposure data^3^** | **Core sample^4^** |
| --- | --- | --- | --- | --- |
|  | *n =* 3,149 | *n =* 4,566 | *n* = 9,223 | *n* = 10,405 |
| Lack of care | 1.12 (0.99, 1.27) | 1.14 (1.02, 1.26) | 1.14 (1.01, 1.29) | 1.14 (1.02, 1.26) |
| Overprotection | 0.99 (0.87, 1.13) | 0.97 (0.87, 1.10) | 0.98 (0.87, 1.10) | 1.01 (0.90, 1.11) |

¹Complete cases;

²Imputed exposure and confounding variables;

^3^Imputed confounding variables and outcome measure;

^4^Imputed exposure, confounding variables and outcome measure
